# Corrigendum to ‘Optimizing care for patients after major anticoagulant-related bleeding: a narrative review and proposal for a clinical care pathway’ [Research and Practice in Thrombosis and Haemostasis 9(7) (2025) 103183]

**DOI:** 10.1016/j.rpth.2025.103243

**Published:** 2025-11-07

**Authors:** Jurjen F. Krommenhoek, Eleonora Camilleri, Thijs E. van Mens, Menno V. Huisman, Nyika D. Kruyt, Ellis S. van Etten, Maarten E. Tushuizen, Serge A.I.P. Trines, Nienke van Rein, Allard Aukema, Frederikus A. Klok, Paul L. den Exter

The authors regret that some author names were incorrectly listed. The names have been corrected online and in the above.

The incorrect names were previously listed as:

Jurjen Krommenhoek^1^, Eleonora Camilleri^2^, Thijs van Mens^1^, Menno Huisman^1^, Nyika Kruyt^3^, Ellis van Etten^3^, Maarten Tushuizen^4^, Serge Trines^5^, Nienke van Rein^2,6^, Allard Aukema^7^, Erik Klok^1^, Paul den Exter^1^

The authors would like to apologise for any inconvenience caused.

